# A zebrafish model of manganism reveals reversible and treatable symptoms that are independent of neurotoxicity

**DOI:** 10.1242/dmm.016683

**Published:** 2014-09-26

**Authors:** Subha Bakthavatsalam, Shreya Das Sharma, Mahendra Sonawane, Vatsala Thirumalai, Ankona Datta

**Affiliations:** 1 Department of Chemical Sciences, Tata Institute of Fundamental Research, 1 Homi Bhabha Road, Colaba, Mumbai-400005, India.; 2 National Centre for Biological Sciences, Tata Institute of Fundamental Research, Bellary Road, Bangalore-560065, India.; 3 Department of Biological Sciences, Tata Institute of Fundamental Research, 1 Homi Bhabha Road, Colaba, Mumbai-400005, India.

**Keywords:** Zebrafish, Manganism, Mechanotransduction, Fictive motor patterns, Dopaminergic neurons

## Abstract

Manganese (manganese ion; referred to as Mn) is essential for neuronal function, yet it is toxic at high concentrations. Environmental and occupational exposure to high concentrations of Mn causes manganism, a well-defined movement disorder in humans, with symptoms resembling Parkinson’s disease (PD). However, manganism is distinct from PD and the neural basis of its pathology is poorly understood. To address this issue, we generated a zebrafish model of manganism by incubating larvae in rearing medium containing Mn. We find that Mn-treated zebrafish larvae exhibit specific postural and locomotor defects. Larvae begin to float on their sides, show a curved spine and swim in circles. We discovered that treatment with Mn causes postural defects by interfering with mechanotransduction at the neuromasts. Furthermore, we find that the circling locomotion could be caused by long-duration bursting in the motor neurons, which can lead to long-duration tail bends in the Mn-treated larvae. Mn-treated larvae also exhibited fewer startle movements. Additionally, we show that the intensity of tyrosine hydroxylase immunoreactivity is reversibly reduced after Mn-treatment. This led us to propose that reduced dopamine neuromodulation drives the changes in startle movements. To test this, when we supplied an external source of dopamine to Mn-treated larvae, the larvae exhibited a normal number of startle swims. Taken together, these results indicate that Mn interferes with neuronal function at the sensory, motor and modulatory levels, and open avenues for therapeutically targeted studies on the zebrafish model of manganism.

## INTRODUCTION

Manganese (manganese ion; referred to here as Mn) plays an important role in neural function by participating in many cellular processes (Aschner and Aschner, 2005). However, chronic exposure to high levels of Mn results in its accumulation in the brain, which in turn leads to a debilitating neurological condition called manganism (Olanow, 2004; Bowman et al., 2011; Racette et al., 2012a; Racette et al., 2012b; Guilarte, 2013). In the previous century, this disease was also known as ‘miner’s disease’ as mining and smelting involves exposure to high levels of Mn dust. However, Mn exposure is not limited to these occupations as environmental and dietary exposure to Mn is on the rise – Mn-containing compounds are added to gasoline to increase its octane rating, to water to disinfect it, as an additive to infant formulas and parenteral nutrition, and several cases of movement disorders resulting from exposure through these routes have been reported (Fell et al., 1996; Kaiser, 2003; Erikson et al., 2007).

Individuals with manganism exhibit motor disturbances resembling Parkinson’s disease (PD), as which it is frequently misdiagnosed. However, the pathology of manganism is distinct from PD in many ways (Olanow, 2004; Bowman et al., 2011). Yet, very little is understood about the neurobiological substrates of Mn intoxication. Previous studies have focused on Mn-induced dopaminergic neurodegeneration and dysfunction using worm (Au et al., 2009; Settivari et al., 2009), rodent (Zhao et al., 2009; Sanchez-Betancourt et al., 2012) and non-human primate models (Burton and Guilarte, 2009). Nevertheless, many questions remain: (1) how does Mn affect neuronal function at dosages far less than those that cause neurodegeneration? (2) What are the effects of Mn on dopaminergic and non-dopaminergic neurons? (3) How can these cellular effects explain the symptoms seen in manganism?

To answer these questions, we developed a larval zebrafish model recapitulating the locomotor deficits that are observed in human manganism. Zebrafish have been used to model many human neurological conditions, such as Alzheimer’s disease, PD and spinal cord injury, and have provided valuable mechanistic insights into these diseases (Kalueff et al., 2014). Larval zebrafish are also widely used in toxicity screens because they have a permeable skin through which substances added in the rearing medium are easily taken up. This allows for greater control over dosage and ease of administering substances to large numbers of animals (Hill et al., 2005). Additionally, larvae show many behaviors, which can be exploited in assays to test the effects of the treatment. Being a vertebrate, the central nervous system of larval zebrafish shows a highly homologous ground plan compared to humans. Therefore, putative targets in humans can be derived from toxicology studies performed in larval zebrafish. Furthermore, neural circuits can be analyzed using a host of techniques, including electrophysiology, immunohistochemistry and imaging (McLean and Fetcho, 2004).

In this study, we show that many of the postural and locomotor phenotypes of human manganism can be replicated in a larval zebrafish model of the disease. Using tissue-labeling methods, high-speed videography and recordings of fictive motor patterns, we show that such phenotypes arise from impaired function of specific sensory, motor and dopaminergic pathways.

**TRANSLATIONAL IMPACT**

**Clinical issue**

Manganese (manganese ion, referred to as Mn) is an essential metal ion that plays multiple roles in the vertebrate system, especially in the brain. Mn is also a component of many industrial processes and has wide applications in welding, smelting, agriculture and water purification, as well as in the transportation sector. Chronic environmental and occupational exposure to Mn can lead to a movement disorder, manganism, with symptoms similar to those of Parkinson’s disease. Owing to these similar symptoms, it often becomes difficult to diagnose and, hence, treat manganism. A major issue leading to misdiagnosis is the lack of knowledge of the neural basis that underlies the symptoms of the pathology. This work set out to identify the neural basis of postural and locomotor defects in manganism by establishing a zebrafish larval model.

**Results**

A zebrafish larval model was generated that recapitulates several of the movement and postural disorders observed in human manganism. This model showed that balance defects arising from Mn exposure are due to functionally impaired hair cells in the larvae. Hearing deficits are prevalent in individuals with manganism, and this work shows that this might be due to the effects of Mn toxicity on hair cells. The study was also able to correlate locomotor disorders to impaired motor function and dopamine dysregulation. Notably, these effects could be partially rescued by removing Mn from the rearing medium. Furthermore, functional impairment of sensory, modulatory and motor neuronal circuits adequately explained several of the observed locomotor defects.

**Implications and future directions**

Chronic Mn exposure is almost unavoidable in the existing urban lifestyle. It therefore becomes very important to elucidate the neuronal basis of manganism and to lay down the foundation for therapeutic interventions. This work developed a zebrafish-based model for manganism that replicates many of the postural and locomotor symptoms of manganism and, hence, will be amenable to testing the effects of therapeutics. Using this model system, the study sketched links between the Mn-exposure-associated symptoms and the affected neuronal regions. The identified neuronal systems can be further probed to guide the development of combative therapeutic routes. The study also indicates that some of the functional effects of chronic Mn exposure are reversible and thus removal of the subject from an Mn-containing environment can offer partial relief from the pathological symptoms at early stages.

## RESULTS

### Mn exposure causes significant morphological and behavioral defects

**Fig. 1.**
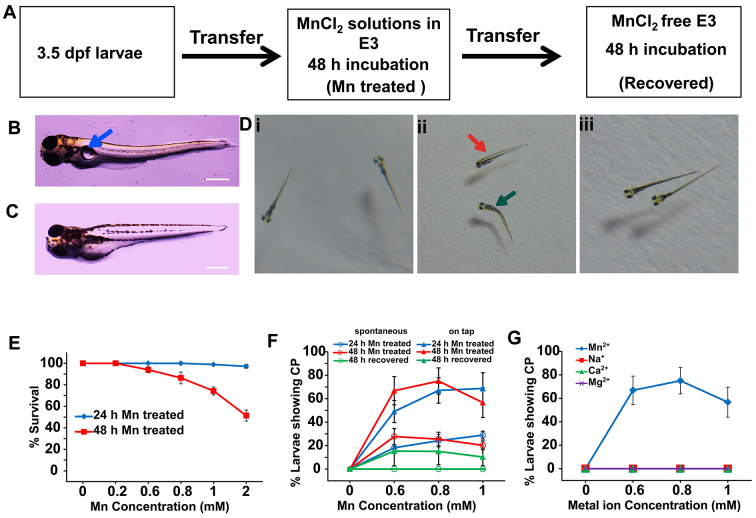
**Postural, morphological and swim defects in Mn-exposed zebrafish larvae.** (A) Scheme representing the treatment protocol. (B) Control 5-dpf larva showing a normal swim bladder (blue arrow). (C) Mn-treated larvae showing an underdeveloped swim bladder. (D) Body position and morphology of free-swimming larvae: (i) control, (ii) Mn-treated and (iii) recovered larvae. Curvature of the spine (green arrow) and sideways floating (red arrow) were observed upon Mn exposure (middle panel). (E) Percentage survival for larvae treated for 24 (blue) or 48 hours (red) with Mn. When larvae were treated with 2 mM MnCl_2_ for 48 hours, 50% survival was observed (*n*=20, six batches). (F) The percentage of live larvae that exhibited spontaneous (circles) and stimulated (triangles) circular swimming patterns (CP) at different levels of Mn exposure. Spontaneous CP completely recovers after removal from Mn-containing medium, whereas ‘on-tap’ CP partially recovers. Statistical analyses were performed by using chi-square test to compare recovered with Mn-treated larvae (*n*=20, three batches). (G) The percentage of live larvae that demonstrated stimulated CP upon 48 hours of exposure to different metal ions: MnCl_2_ (blue), CaCl_2_ (green), NaCl (red) and MgCl_2_ (purple). Error bars in the plots represent s.e.m.

Chronic Mn exposure in vertebrates induces locomotion and balance defects (Au et al., 2009; Bowman et al., 2011; Criswell et al., 2012). To elucidate the neuronal basis of Mn-induced motion defects, we reared zebrafish larvae from 3.5 days post fertilization (dpf) in E3 medium containing varying concentrations of MnCl_2_, as this salt is water soluble. The larvae were treated for 24 to 48 hours with MnCl_2_, and these larvae will be henceforth referred to as Mn-treated larvae (Fig. 1A). Untreated larvae will be referred to as controls. In order to test the effect of the removal from Mn-containing medium, the Mn-treated larvae were allowed to recover in Mn-free medium for 24 to 48 hours following the initial treatment with Mn (Fig. 1A). These larvae will be referred to as recovered larvae.

We determined the 50 percent lethal concentration (LC_50_) value to be 2 mM of externally added MnCl_2_ (48-hour exposure, Fig. 1E, red curve). Therefore, in all subsequent experiments, we used concentrations below 2 mM to assess the behavioral effects of Mn exposure.

Within 24 hours of exposure to 1 mM MnCl_2_, the Mn-treated larvae showed visible defects, including an underdeveloped swim bladder (Fig. 1B,C). The larvae displayed balance defects characterized by a tendency to float sideways (Fig. 1Dii), and sometimes upside down. The percentage of live Mn-treated larvae (48-hour exposure to 1 mM MnCl_2_) showing sideways floating was found to be 98±2% (three batches of *n*=20 larvae). None of the control larvae (three batches of *n*=20 larvae) showed sideways floating (Fig. 1Di). Several Mn-treated larvae displayed postural defects, which included larvae with a curved spine (Fig. 1Dii, green arrow). The percentage of live Mn-treated larvae (48-hour exposure to 1 mM MnCl_2_) with a curved spine was 65±2%, whereas the control larvae did not exhibit curved spines and had straight trunks (Fig. 1Di, three batches of *n*=20 larvae).

In Mn-treated larvae, a distinct circular swimming pattern was observed in both spontaneous movements and in response to vibrational stimuli (supplementary material Movie 1). Clockwise and anti-clockwise circular swimming, looping and corkscrew-like swimming were characteristic of the circular swimming pattern. Movements were, in general, slower and less frequent in the Mn-treated larvae, and the fish had a tendency to remain at the bottom of the wells. To better characterize the circular swimming pattern, we grouped larvae into six batches of 20 larvae (at 3 dpf), each in concentrations of MnCl_2_ ranging from 0 to 1 mM. The larvae were observed after 24 and 48 hours of Mn exposure and scored as either presenting or not presenting a circular swimming pattern. We found that larvae exhibited a circular swimming pattern at MnCl_2_ doses of 0.6 mM and above. We then calculated the percentage of larvae showing circular swimming patterns spontaneously and in response to vibrational stimuli (Fig. 1F, red and blue traces). When the Mn-treated larvae (48-hour MnCl_2_ exposure) were placed back into Mn-free E3 medium for 48 hours, the spontaneous circular swimming pattern was completely abolished, whereas the ‘on-tap’ circular swimming pattern was significantly reduced [Fig. 1F, green traces; *P*<0.0001, degrees of freedom (df)=5, *n*=20 larvae, three batches]. The larvae also started floating and swimming normally, and the curved spine became straight in several cases (Fig. 1Diii). The percentage of larvae displaying sideways floating was 12±2% in the recovered larvae (48-hour exposure to 1 mM MnCl_2_ followed by a 48-hour recovery period in Mn-free E3 medium, calculated for three batches with 20 larvae in each) versus 98±2% of Mn-treated larvae (*P*<0.0001, df=3). Similarly, the percentage of larvae exhibiting curved spines was 27±4% in the recovered larvae (calculated for three batches of 20 larvae each) versus 65±2% of Mn-treated larvae (*P*<0.0001, df=3).

None of the structural, morphological or behavioral changes seen in Mn-treated larvae were observed when they were exposed to other biologically essential monovalent and divalent metal ions. In the case of Ca^2+^, high concentrations above 0.6 mM induced a fast tail wagging. Impaired Cu^2+^ and Zn^2+^ homeostasis have been implicated in neurological disorders, such as Alzheimer’s disease (Breydo and Uversky, 2011); hence, the effects of these metal ions were also monitored. Cu^2+^ and Zn^2+^ were lethal to the larvae at high concentrations (>50 μM for Cu^2+^ and >200 μM for Zn^2+^); however, the larvae did not exhibit any distinct behavioral or structural defects at lower concentrations (<100 μM). We monitored larvae that had been treated with Na^+^, Ca^2+^ and Mg^2+^ for circular swimming patterns and found that none of these ions induce such behavior (Fig. 1G). The results of these metal ion exposure studies together with the recovery data on the Mn-exposed larvae clearly indicate the specificity of Mn in producing morphological, postural and locomotor disorders.

### Mn suppresses mechanotransduction

**Fig. 2.**
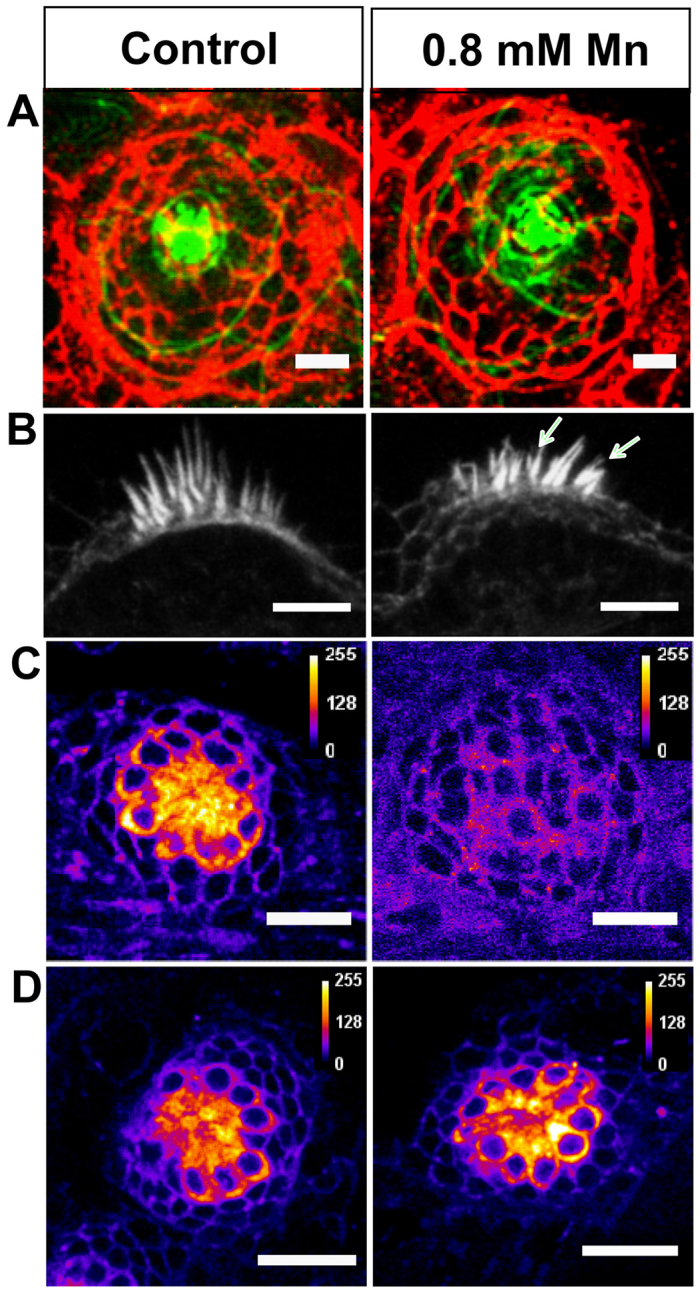
**Effect of Mn exposure on neuromast structure and function.** (A) Confocal images of a representative neuromast in the anterior lateral line of control and Mn-treated (48-hour exposure) larvae. Cell membranes immunostained for E-cadherin (red), and actin filaments stained with phalloidin (green) are depicted as a merged image that was generated by choosing *z* planes with maximum intensities for each dye. (B) Confocal images of representative otic neuromasts highlighting splayed stereocilia (arrows) in an Mn-treated larva. (C,D) Confocal images of a representative neuromast in the anterior lateral line of larvae stained with FM 1-43 FX dye. Images indicate a particular *z* plane at which maximal staining was observed for control (C and D, left), Mn-treated (48-hour exposure) (C, right) and recovered (48 hours in Mn-free medium following Mn exposure) (D, right) larvae. FM-dye uptake was suppressed upon Mn exposure. Scale bars: 25 μm (A,C,D); 5 μm (B).

We found that zebrafish larvae exposed to Mn displayed postural and balance defects (Fig. 1Dii). In aquatic vertebrates, balance is mediated by neuromasts, which are mechanosensory organs containing hair cells. Defective neuromasts have been associated with balance and postural defects in larval systems (Nicolson et al., 1998). We therefore imaged neuromasts in untreated and Mn-treated larvae. Larvae were stained with an antibody against E-cadherin, which allowed visualization of cell membranes. To check the integrity of stereocilia, the actin bundles were co-stained with Alexa-Fluor-488-conjugated phalloidin. The neuromast clusters, when viewed from above, appear as flower-shaped structures or rosettes, with the stereocilia in the center. Fig. 2A shows confocal images of neuromasts in the head region (anterior lateral line) of control and Mn-treated larvae. On average, both Mn-treated and control larvae had 19±1 neuromasts (*n*=4 for both control and Mn-treated larvae upon a 48-hour exposure to 1 mM MnCl_2_), indicating that the number of neuromasts was conserved even after Mn exposure (*P*=1). To further investigate the effect of treatment with Mn on the integrity of neuromasts, the number of hair cells per neuromast was counted within the MI2 and IO4 neuromasts in the anterior lateral line of both control and Mn-treated larvae (*n*=5 for each). The number of hair cells in the selected neuromasts was 7±1 in control and 8±1 in Mn-treated fish, indicating that Mn did not affect the hair cell count within the neuromast (*P*=0.7507). However, the stereociliary bundles in the otic neuromasts when viewed from the side appeared splayed in the Mn-treated larvae (43%, 101 bundles, *n*=9, Fig. 2B) unlike in the control larvae, which showed mostly intact bundles (0.6% splayed, 75 bundles, *n*=6, Fig. 2B).

To test whether neuromast function was affected by exposure to Mn, we used a styryl dye FM1-43 that selectively labels hair cells. FM1-43 is taken up through open ion channels in neuromasts, and dye uptake indicates that the mechanosensory apparatus is functionally intact (Seiler and Nicolson, 1999; Gale et al., 2001; Meyers et al., 2003). Our analysis revealed that untreated larvae showed bright fluorescence (Fig. 2C) throughout the rosette structure of the neuromast, whereas Mn-treated larvae showed negligible fluorescence (Fig. 2C). When we transferred the Mn-treated larvae back into normal E3 medium for 48 hours, we observed that the FM1-43 staining was rescued (Fig. 2D). This result demonstrates that mechanotransduction in larval neuromasts is impaired upon Mn exposure. Importantly, this defect was reversible and was rescued by removing Mn from the rearing medium.

### High-speed videography reveals locomotor deficits in Mn-treated larvae

**Fig. 3.**
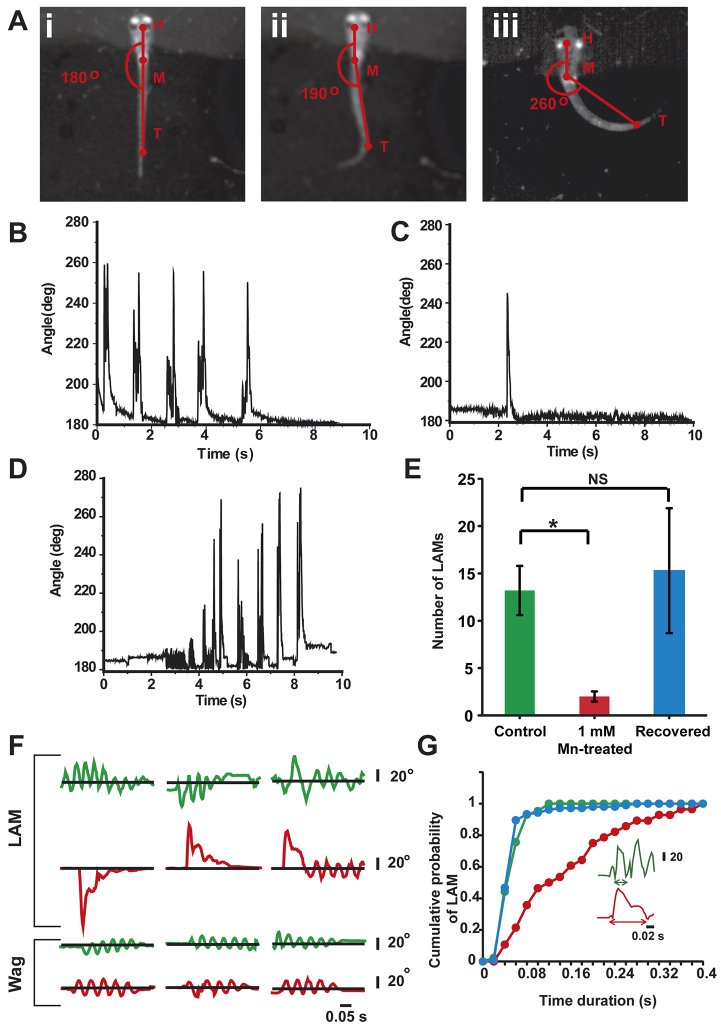
**Altered frequency and duration of trunk movements in Mn-exposed larvae.** (A) Still frames from a 20-second movie recorded at 300 fps in a head-embedded larva. The angle between head ‘H’, mid trunk ‘M’ and tail ‘T’ was used as a measure of the extent of trunk movement when the larva was exhibiting (i) a resting state (180°), (ii) wagging (<200°, supplementary material Movie 2) and (iii) large-angle movements (LAM) (>200°, supplementary material Movies 3, 4). The trunk movement angle plotted against time for (B) control larvae, (C) Mn-treated (48-hour exposure to 1 mM MnCl_2_) and (D) recovered (24-hour recovery following Mn exposure) larvae. (E) Average number of LAMs within a 20-second period plotted for control (*n*=5), Mn-treated (*n*=7) and recovered (*n*=7) larvae. Error bars indicate s.e.m. Statistical analysis was performed by using Wilcoxon rank sum test with Bonferroni correction for multiple comparisons. *P=0.0025; NS, non significant. (F) Stacks of expanded plots of LAMs and short wags in control (green) and Mn-exposed larvae (red). The black line represents the resting position. Angles above the line depict left-directed movements and angles below the line depict right-directed movements. Alternate consecutive left and right LAM patterns, and clusters of unidirectional LAMs were observed in control larvae, whereas Mn-exposed larvae exhibited single LAMs which were not immediately followed by other LAMs. (G) Cumulative probability distribution of LAM durations for control (green), Mn-treated (48-hour exposure, red) and recovered (24 hours in Mn-free medium following Mn exposure, blue) larvae indicating that control and recovered larvae displayed shorter duration LAMs, whereas Mn-treated larvae displayed longer duration LAMs (*P*<0.0001). Statistical analysis was performed using Kruskal–Wallis ANOVA.

In human manganism, chronic Mn exposure leads to symptoms resembling PD, such as poorly coordinated and slowed movements, and tremors (Crossgrove and Zheng, 2004; Roth, 2009). When we exposed zebrafish larvae to 1 mM MnCl_2_ for 24 to 48 hours, we observed that they exhibited reduced and slow movements, as well as a circular swimming pattern (Fig. 1E; supplementary material Movie 1). We wanted to test whether these locomotor deficits were due to the direct effects of Mn on motor networks or whether they resulted from the balance defects. For these investigations, we embedded larvae in agarose in a dorso-ventral position in order to balance them, and we then removed the agarose surrounding the tail so that it was free to move. In this configuration we recorded the tail beats in response to vibrational stimuli at 300 frames per second (fps) and extracted the frequency and amplitude of tail beats from these videos. The amplitudes of the tail beats were reported as body angles, where a body angle close to 180° was defined as the resting position of the larvae (Fig. 3Ai). We observed low amplitude tail beats (‘wags’; body angles <200°, Fig. 3Aii; supplementary material Movie 2), as well as large-angle movements (‘LAMs’; body angles >200° and <270°) of the tail (Fig. 3Aiii; supplementary material Movies 3, 4). The short tail wags occurred in bursts, and several wags were clustered together. The LAMs were intermittent and interspersed between the wags. Over a 20-second duration of recording, we observed that the Mn-treated larvae showed fewer LAMs compared with untreated larvae (Fig. 3C,B, respectively; control 13.2±2.6, *n*=5; Mn-treated 2±0.5, *n*=7; *P*=0.0025). The number of LAMs increased and was similar to that of control when the larvae were allowed to recover in Mn-free medium for 24 hours (Fig. 3D,E; recovered 15.3±6.60, *n*=7, *P*=0.72). When we examined each LAM in more detail, we observed that in control larvae, alternating left and right tail beats, as well as unidirectional tail beats (Fig. 3F), occurred in quick succession, whereas in Mn-treated larvae, single unidirectional tail beats of long durations were present (Fig. 3F). The LAMs were of significantly longer duration in Mn-treated larvae compared with those of untreated and recovered larvae (Fig. 3G; control 57±3 mseconds, *n*=5; Mn-treated 163.4±23 mseconds, *n*=7; recovered 54.7±4 mseconds, *n*=7; *P*<0.0001). However, the short tail wags were similar in duration in Mn-treated and untreated larvae (Fig. 3F; control larvae 39.8±1.8 mseconds; Mn-treated 36.9±1.9 mseconds; *P*=0.34), suggesting that treatment with Mn induces specific impairments in some locomotor behaviors and not others. These data demonstrate that in addition to impaired mechanotransduction leading to postural abnormalities, Mn-treated larvae also exhibit impaired swim motor patterns.

### Mn does not affect motor neuronal innervation of muscles

**Fig. 4.**
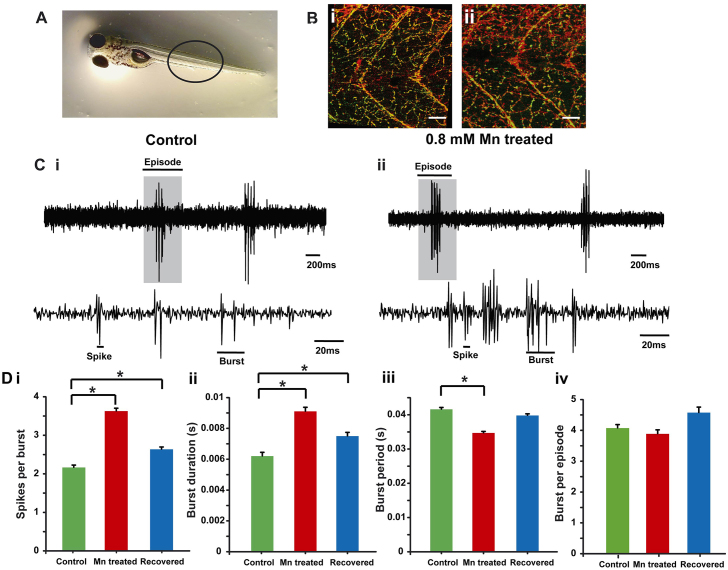
**Morphology and function of motor neurons.** (A) Zebrafish larvae, the region where ZNP-1 antibody and α-bungarotoxin staining was visualized (black circle) is highlighted. (B) A single optical slice showing ZNP-1 staining (red) and α-bungarotoxin staining (green, colocalized as yellow) of motor neuron innervation of axial muscles in (i) control and (ii) Mn-treated (48-hour exposure) larvae. The staining indicates similar motor neuronal axon morphology between the two groups. Scale bars: 30 μm. (C) Extracellular recordings of fictive swim patterns on motor neurons highlight that episodes comprise bursts and spikes in (i) control and (ii) Mn-treated larvae. The top traces show episodes of swim motor patterns. The episode highlighted by a gray box is shown expanded in the bottom traces. (D) Bar plots for (i) the number of spikes per burst, (ii) the burst duration, (iii) the burst period and (iv) the number of bursts per episode in control (green), Mn-treated (red) and recovered (blue) larvae. Data represent mean±s.e.m. *Values that are significantly different from control at an α level of 0.05 as estimated by using the Kruskal–Wallis test.

High-frequency tail beats of large amplitudes were not observed in our high-speed videography recordings of Mn-treated larvae. This could result from a loss of motor neuronal innervation of muscles upon treatment with Mn. To test this prediction, we immunostained motor axons using α-bungarotoxin and the ZNP-1 antibody (against Synaptotagmin-2) in untreated and Mn-treated larvae. We found that muscle segments (Fig. 4A) were equally well-innervated in untreated and Mn-treated larvae (Fig. 4Bi,ii).

### Fictive motor patterns are altered upon Mn treatment

Next, we wanted to test whether the swim central pattern generator (CPG) was affected due to Mn exposure. In spite of normal innervation of the musculature, abnormal firing of motor neurons can lead to altered kinematics of swimming. To test this, we recorded multiunit motor neuronal spikes in paralyzed larvae by using suction electrodes applied to their nerve-muscle junctions and analyzed the temporal structure of these motor patterns. In both control and Mn-treated larvae (48-hour exposure to 0.8 mM MnCl_2_), we observed motor neuronal bursts that occurred in an episodic fashion (Fig. 4Ci,ii), with each burst corresponding to one unilateral tail bend (Masino and Fetcho, 2005). We saw that Mn-treated larvae had more spikes in each burst compared with control larvae (Fig. 4Ci,ii, lower traces, Fig. 4Di).The duration of each burst was longer (Fig. 4Dii) as a result of the increase in the number of spikes per burst, whereas the cycle period of bursts was decreased (Fig. 4Diii). The number of bursts generated during each episode of the motor pattern was unaltered (Fig. 4Div). When Mn-treated larvae were placed in normal saline and allowed to recover for 48 hours, the cycle period returned to normal. The burst duration and the number of spikes per burst, however, continued to be significantly higher than those of control larvae (Fig. 4Di,ii). These data suggest that the motor pattern elicited after treatment with Mn is substantially different from that generated in control larvae and that some changes to the motor pattern are long lasting and persist even after Mn is removed from the rearing medium.

### Treatment with Mn gives rise to defects in the dopaminergic circuitry

**Fig. 5.**
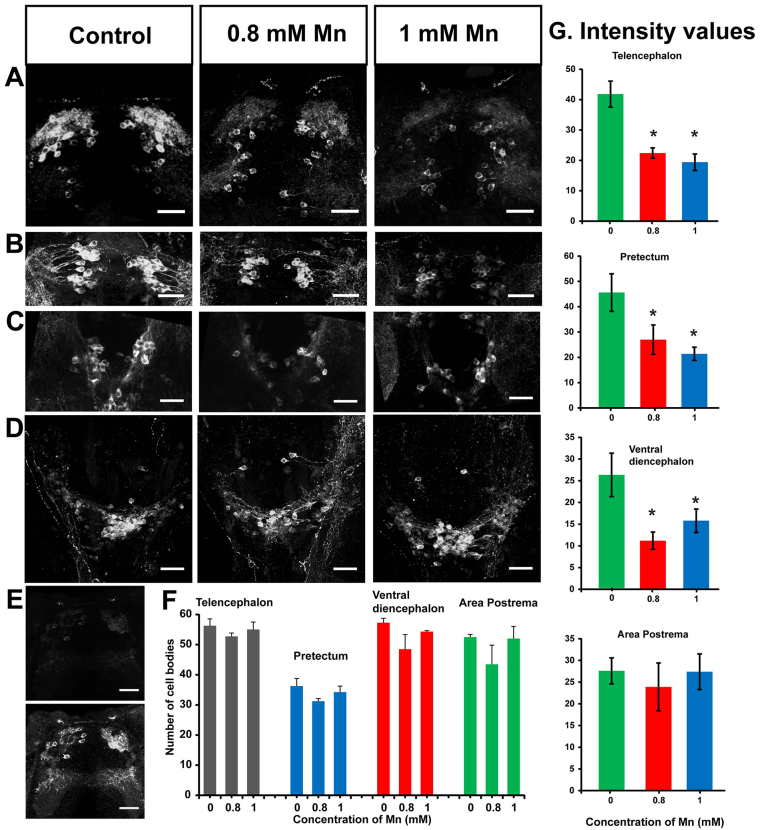
**Reduction in tyrosine hydroxylase immunoreactivity upon Mn exposure.** (A–D) Confocal images of catecholaminergic neurons comprising of dopaminergic and noradrenergic neurons in 5-dpf larvae (dorsal view) stained with an antibody against tyrosine hydroxylase showing a reduction in staining upon 48 hours of exposure to Mn. Maximum intensity projection (*z*-axis) images of tyrosine hydroxylase immunoreactivity in (A) telencephalon, (B) pretectum, (C) ventral diencephalon and (D) area postrema. (E) A single optical slice showing tyrosine hydroxylase immunoreactivity in the telencephalon of an Mn-exposed larva before (top) and after (bottom) image intensity multiplication. Scale bars: 30 μm. (F) The number of tyrosine-hydroxylase-immunoreactive cell bodies in different regions of 5-dpf larvae exposed to the indicated concentrations of Mn showed no significant differences in cell counts (*n*=4; telencephalon *P*=0.50; pretectum *P*=0.23; ventral diencephalon *P*=0.057 for control and those treated with 0.8 mM Mn, and *P*=0.23 for control and those treated with 1 mM Mn; and area postrema *P*=0.31). Error bars represent s.e.m. Statistical tests were performed using ANOVA and Wilcoxon rank sum test with Bonferroni correction for multiple comparisons. (G) Bar plots indicating the average intensities of tyrosine hydroxylase immunostaining in control larvae and Mn-treated larvae from identical ROIs drawn in the regions indicated on top of each plot. Error bars represent s.e.m. RMANOVA and Freidman tests were performed to calculate statistical significance. *Values that are significantly different from control at an α level of 0.05.

Our findings thus far indicate that the kinematics of movements induced by vibrational stimuli are affected by Mn exposure; larvae generate fewer and longer-duration large angle tail bends; and several parameters of the swim motor pattern, including the burst durations and burst period, are altered as a result of treatment with Mn. The duration of bursts is governed by motor neuronal intrinsic conductances and pre-motor synaptic drive, which may themselves be the target of descending neuromodulation. Dopamine is a candidate neuromodulator that has been shown to affect motor neuronal bursting in zebrafish and other animals (Harris-Warrick et al., 1998; Whelan et al., 2000; Svensson et al., 2003; Thirumalai and Cline, 2008; Lambert et al., 2012). Here, we probed whether the dopaminergic circuits in Mn-treated (48-hour exposure to 1 mM MnCl_2_) larvae were intact or compromised, by staining with an antibody against tyrosine hydroxylase (Fig. 5A–D). In untreated zebrafish larvae, dopaminergic cells are found in several nuclei distributed across the rostro-caudal extent of the central nervous system (CNS), including the telencephalon, the pretectum, ventral diencephalon and the area postrema of the hindbrain (Rink and Wullimann, 2002; McLean and Fetcho, 2004). The latter two clusters are mixtures of noradrenergic and dopaminergic cell groups, both of which are recognized by the antibody used. Intensity analysis of the images was performed to quantify tyrosine hydroxylase immunoreactivity in each cluster. The analysis indicated that the staining was significantly reduced in larvae that had been treated with 0.8 mM or 1 mM MnCl_2_ (48 hour exposure) in the telencephalon, pretectum and ventral diencephalon regions (*P*=0.011, 0.006 and 0.022 respectively, *n*=6) compared with control larvae (Fig. 5G). The area postrema region did not show any significant change in the intensity of staining in larvae that had been treated with either 0.8 mM or 1 mM Mn (*P*=0.604, *n*=6, Fig. 5G) when compared with that of control larvae. Considering that the staining and imaging were performed under the same conditions and in parallel for the two groups, our results indicate a decrease in tyrosine hydroxylase immunoreactivity upon treatment with Mn in all catecholaminergic neurons analyzed, except for those in the area postrema. This further suggests that tyrosine hydroxylase expression levels are reduced when larvae are exposed to MnCl_2_.

To further investigate whether treatment with Mn results in a loss of dopaminergic neurons, as happens in PD, we counted the number of stained cells in each of these cell clusters. For this analysis, we multiplied the intensity of the images from Mn-treated larvae by a constant number to detect the presence of the neurons (Fig. 5E). We found that the number of tyrosine-hydroxylase-positive cells was not altered upon treatment with Mn in any of the cell groups (Fig. 5F; telencephalon *P*=0.50; pretectum *P*=0.23; ventral diencephalon *P*=0.057 for control and those treated with 0.8 mM Mn, and *P*=0.23 for control and those treated with 1 mM Mn; and area postrema *P*=0.31, *n*=4).

### The effect of Mn on tyrosine hydroxylase immunoreactivity is reversible

**Fig. 6.**
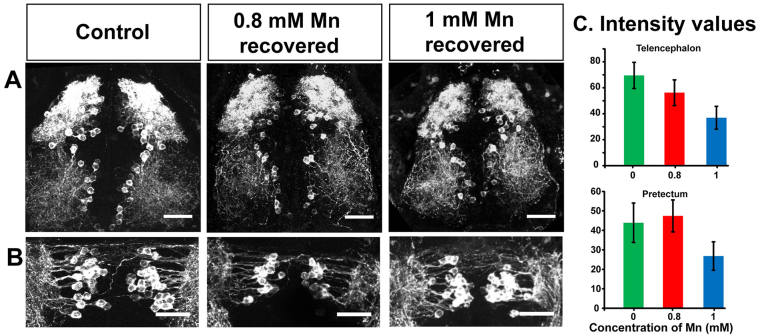
**Recovery of tyrosine hydroxylase immunoreactivity.** (A,B) Tyrosine hydroxylase immunoreactivity recovers when Mn is removed from the rearing medium. Confocal maximum intensity projection (*z*-axis) images of catecholaminergic neurons of control and recovered larvae (dorsal view) stained with an antibody against tyrosine hydroxylase depicting the (A) telencephalon and (B) pretectum regions. Scale bars: 30 μm. (C) Bar plots representing the average intensity of tyrosine hydroxylase immunostaining in recovered larvae compared to that of control larvae from identical ROIs drawn in the regions indicated at the top of each plot. Error bars represent s.e.m. RMANOVA was used to calculate statistical significance. *P*=0.0908 and 0.2002 for telencephalon region and prectum region, respectively.

Finally, we tested whether the reduction in the intensity of tyrosine hydroxylase staining that we observed after treatment with Mn could be recovered if larvae were transferred into Mn-free E3 medium for 48 hours following Mn exposure. We fixed these larvae and processed them for tyrosine hydroxylase immunoreactivity (Fig. 6A,B). We found that the intensity of tyrosine hydroxylase immunoreactivity in larvae that had been previously treated with 0.8 mM or 1 mM MnCl_2_ preceding removal from the Mn-containing medium, was similar to that of untreated larvae in the telencephalon and pretectum regions (*P*=0.0908 and *P*=0.2002, respectively, *n*=7, Fig. 6C). These results suggest that the Mn-induced reduction in dopaminergic drive, as reflected by the lowering of tyrosine hydroxylase levels, could mediate the reversible deficits in swimming behavior that we observed.

### Dopamine rescues the frequency of large-angle tail beats in Mn-treated larvae

**Fig. 7.**
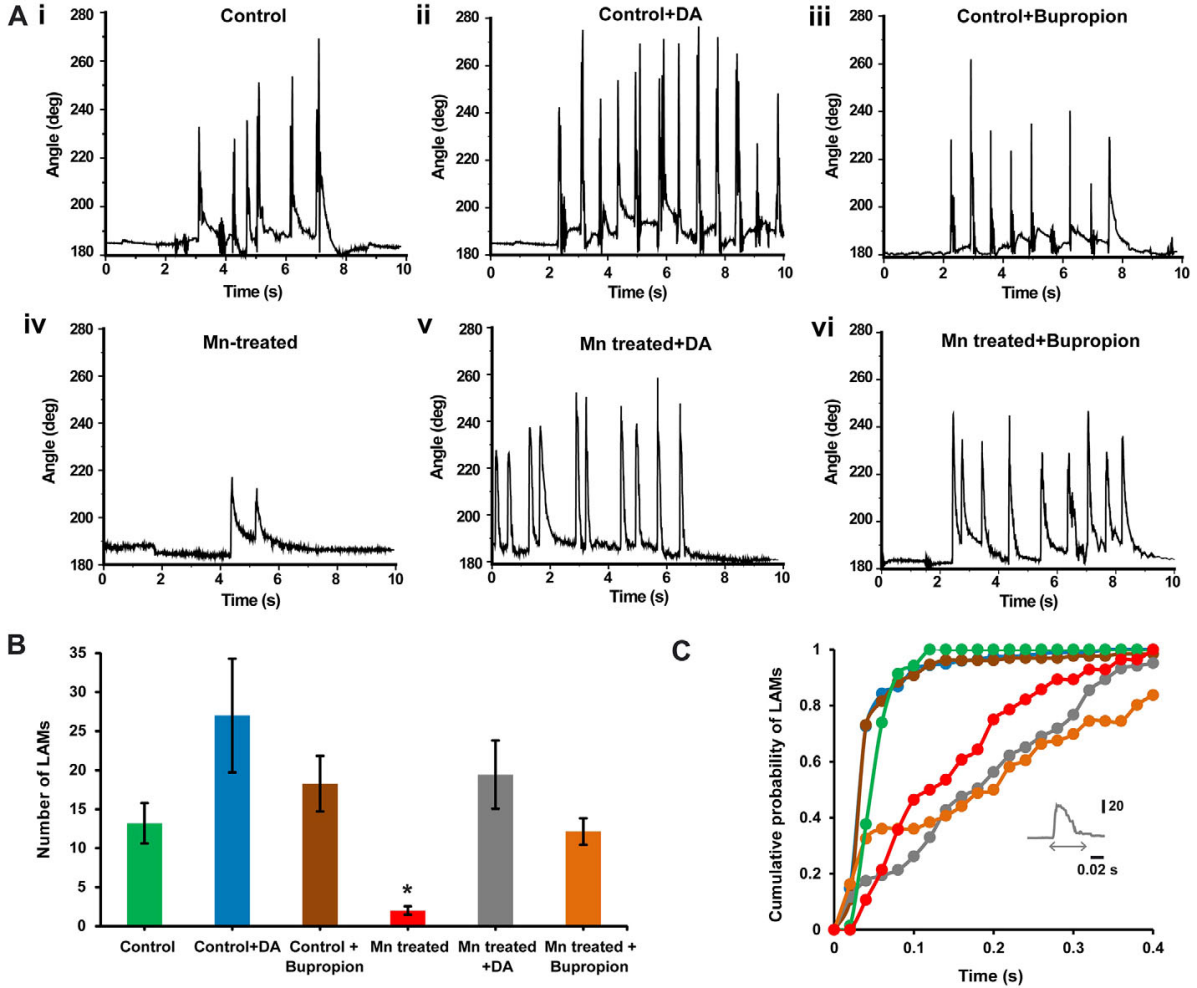
**Treatment with Mn might alter LAMs by affecting dopaminergic drive.** (A) The trunk movement angle plotted against time for (i) control (ii) dopamine (DA)-supplemented control, (iii) bupropion-supplemented control, (iv) Mn-treated (48-hour exposure to 1 mM MnCl_2_), (v) Mn-treated dopamine-supplemented (48-hour exposure to 1 mM MnCl_2_ followed by supplementation with dopamine) and (vi) Mn-treated bupropion-supplemented (48-hour exposure to 1 mM MnCl_2_ followed by supplementation with bupropion) larvae. (B) The average number of LAMs within a 20-second time period plotted for control (*n*=5), dopamine-supplemented control (*n*=7), bupropion-supplemented control (*n*=7), Mn-treated (*n*=7), Mn-treated dopamine-supplemented (*n*=7) and Mn-treated bupropion-supplemented (*n*=7) larvae. Error bars indicate s.e.m. Statistical tests were performed by using the Wilcoxon rank sum test with Bonferroni correction for multiple comparisons. *Values that are significantly different from control. (C) Cumulative probability distribution of LAM durations for control (green), Mn-treated (red), control dopamine-supplemented (blue), control bupropion-supplemented (brown), Mn-treated dopamine-supplemented (gray) and Mn-treated bupropion-supplemented (orange) larvae.

We tested the hypothesis outlined above by supplementing dopamine or bupropion to Mn-treated larvae. We investigated whether the defects in the number and duration of LAMs that we observed in Mn-treated larvae could be rescued by enhancing the availability of dopamine. Bupropion blocks dopamine re-uptake and increases systemic dopamine levels. The control dopamine-and control bupropion-supplemented larvae displayed similar numbers of LAMs when compared with control larvae in normal medium (Fig. 7Ai–iii,B; *P*=0.41 for control with dopamine, *n*=7; and *P*=0.36 for control with bupropion, *n*=7). As mentioned above, Mn-treated larvae exhibited fewer LAMs than control larvae (Fig. 7Aiv,B, *P*=0.0025). When Mn-treated larvae were placed in medium containing 10 μM dopamine, the average number of LAMs was similar to that of control larvae (Fig. 7Av,B, *n*=7, *P*=0.41). This proved that dopamine successfully rescued the frequency of LAMs in Mn-treated larvae. This result was further confirmed by exposing the Mn-treated larvae to bupropion. The average number of LAMs for the bupropion-supplemented Mn-treated larvae was similar to that of control larvae (Fig. 7Avi,B, *n*=7, *P*=0.77). However, the addition of dopamine or bupropion did not rescue the effect of Mn on the duration of LAMs (194.7±14 mseconds for fish treated with Mn and dopamine; 237.2±22 mseconds for fish treated with Mn and bupropion). The duration of the LAMs continued to be significantly longer compared with those of control, even after supplying dopamine or bupropion to Mn-treated larvae (*P*<0.0001; Fig. 7C). These data indicate that treatment with Mn affects LAM frequency through a dopamine-dependent pathway. However, treatment with Mn results in increases of LAM duration through pathways that do not involve dopaminergic drive.

## DISCUSSION

Chronic exposure to Mn results in a cluster of psychiatric and locomotor disturbances collectively called manganism. The psychiatric symptoms precede the motor symptoms, which are Parkinson-like (Bowman et al., 2011). At the cellular level, Mn exposure leads to mitochondrial dysfunction, release of reactive oxygen species, altered neurotransmitter metabolism and release and eventual cell death (Martinez-Finley et al., 2013). Similar to PD, neurodegeneration has been documented in post-mortem brains of manganism patients and therefore may be a feature of the terminal pathological stage and may not be related to early symptoms (Perl and Olanow, 2007). Thus, in order to develop early-stage combative therapeutic interventions, it is important to understand the subtler effects of Mn toxicity on neural function rather than focusing on cell death alone.

### Treatment with Mn and mechanotransduction

In this study we used a sub-lethal dose of Mn to study how treatment with Mn affects neuronal circuits by using a larval zebrafish model. We observed several locomotor and postural defects, including sideways floating, curvature of the spine, underdevelopment of the swim bladder and, most importantly, a circular swimming pattern. Such phenotypes have been previously observed in zebrafish auditory and vestibular mutants, which have either structurally or functionally defective neuromasts (Nicolson et al., 1998). We therefore hypothesized that defective neuromasts could be an important link between treatment with Mn and the observed circular swimming patterns and postural defects. Importantly, Mn exposure in humans leads to an increased propensity to fall backwards and to postural instability (Bowman et al., 2011), indicating the impairment of the vestibular system. Whether overexposure to Mn causes degeneration of cells in the human vestibular system has not yet been verified. Although hearing deficits have been reported in few manganism cases, it could be an effect of noise exposure or combined noise and Mn exposure (Josephs et al., 2005; Bouchard et al., 2008). *In vitro* studies of Mn exposure on cochlear cultures from postnatal rats have shown that Mn can damage sensory hair cells, auditory nerve fibers and the spiral ganglionic neurons (Ding et al., 2011).

Our zebrafish model recapitulates many of the postural and locomotor deficits seen in human manganism. The observed symptoms are evident in the absence of neurodegeneration in the mechanosensory neuromasts or within the dopaminergic nuclei of the brain. The effect of Mn on mechanosensory hair cells is substantially different from the effects of other transition metals which cause neuromast degeneration (Linbo et al., 2006; Froehlicher et al., 2009). This argues that the postural defects seen upon treatment with Mn are not related to cell death but could result from a structural or functional impairment. Consistent with this, we found splayed stereocilia in the otic neuromasts. Because the stereocilia are the sites of specialization for mechanotransduction (Nicolson et al., 1998) and their tip links are postulated to gate mechanosensory channels, it was necessary to check the functionality of the neuromasts. We observed defective FM1-43-dye loading in neuromasts after Mn exposure. Although initially it was believed that FM1-43 entered hair cells by using apical endocytosis, multiple studies now show that dye loading of hair cells is primarily through direct entry via mechanotransduction channels (Seiler and Nicolson, 1999; Meyers et al., 2003). Absence of FM1-43-dye loading after treatment with Mn indicates that mechanotransduction is impaired in these larvae. It could be that Mn binds to and blocks conduction through these channels, leading to loss of mechanosensation. This is also supported by our recovery experiments, where dye labeling of neuromasts is restored when Mn is removed from the rearing medium. Indeed, although Mn is permeable through many transient receptor potential (TRP) channels involved in mechanotransduction, it has also been shown to block other types of TRP channel, such as the TRPC3 (Streifel et al., 2013). Whether such a blockade of sensory transduction leads to the reported hearing loss and balance defects reported in human manganism requires investigation.

### Treatment with Mn and the swim CPG

To tease apart the neural mechanisms underlying the Mn-induced circular swimming pattern, we took a two-pronged approach: we observed the swimming behavior using high-speed video recording when larvae were embedded in agarose and thus posturally balanced, and secondly we recorded fictive motor patterns occurring spontaneously in paralyzed larvae. The high-speed video recordings clearly showed long unilateral bends of the tail in Mn-treated larvae (Fig. 3F). Furthermore, although the LAMs alternated between the left and right sides in control animals, they were unilateral, tending to only one side, in Mn-treated larvae. In freely swimming larvae, such long unilateral tail bends could result in the circular swimming pattern that we observed. Concomitantly, treatment with Mn increased burst durations and the number of spikes per burst, and decreased the burst period. These changes in the fictive motor pattern are likely to extend the contraction phase of the muscle during one swim cycle. When taken together, our data on the circular swimming pattern, swimming kinematics and motor patterns seem to suggest that Mn alters the firing behavior of motor neurons, which leads to longer contraction phases to one side, which in turn might contribute to the generation of the circular swimming pattern.

When Mn-treated larvae were allowed to recover in Mn-free normal rearing medium, the spontaneous circular swimming pattern was absent, yet a circular swimming pattern could be evoked by tapping, indicating that, despite restoring mechanotransduction (Fig. 2D), the larval CNS was unable to generate appropriate movement in response to vibrational stimuli. This pointed to persistent effects of Mn within the brain and spinal cord, which might manifest as altered motor patterns. Consistent with this, we observed that the number of spikes per burst and the burst duration do not return to control levels after recovery (Fig. 4Di,ii). However, the burst period does return to control values after recovery (Fig. 4Diii). These data indicate that in the spinal circuitry, some properties of motor neuronal firing undergo long-lasting changes after Mn exposure, whereas other properties are only affected as long as Mn is present in the medium.

The increased number of spikes per burst and the burst durations are likely to occur due to direct effects of Mn on motor neuron excitability, or indirectly by affecting the premotor networks of the spinal cord and the brain. As motor neurons are especially vulnerable to oxidative damage, a sub-lethal dose of Mn might affect their neurophysiology by affecting cellular metabolism. Indeed, several cases of Mn smelters and miners exhibiting symptoms of manganism and amyotrophic lateral sclerosis (ALS) have been reported (Bowman et al., 2011). Furthermore, high levels of Mn have been found in the cerebrospinal fluid and postmortem spinal cord cross sections of individuals with ALS (Roos et al., 2012), suggesting that the motor neuron pathology in ALS could be related to Mn toxicity. In future experiments, we hope to decipher the direct effects of Mn on motor neuron physiology.

### Treatment with Mn and dopaminergic neuromodulation

When treated with Mn, zebrafish larvae showed decreased tyrosine hydroxylase immunoreactivity in almost all catecholaminergic nuclei, except the area postrema of the hindbrain. This is consistent with previous *in vitro* studies reporting a decrease in tyrosine hydroxylase immunoreactivity upon Mn exposure (Sriram et al., 2010; Zhang et al., 2011). We did not observe any neurodegeneration of catecholaminergic neurons. Concomitant with the decrease in tyrosine hydroxylase immunoreactivity, Mn-treated larvae also showed a decreased number of LAMs. When Mn-treated larvae were placed in normal rearing medium, the number of LAMs, as well as the tyrosine hydroxylase immunoreactivity, recovered to normal levels. Furthermore, when Mn-treated larvae were acutely treated with either dopamine or the dopamine re-uptake blocker bupropion, the number of LAMs recovered to normal levels. These data indicate that the ability of the larval CNS to initiate LAMs is controlled by dopaminergic drive. LAMs are triggered by the activation of the Mauthner array through lateral line and auditory inputs. An earlier study has shown that the Mauthner neurons and the eighth nerve auditory afferent synapses impinging on them are modulated by dopaminergic inputs from the caudal hypothalamus (Mu et al., 2012). Our results are consistent with this finding and suggest that Mn impairs dopaminergic modulation of the Mauthner network. However, neither addition of dopamine nor bupropion could rescue the duration of the LAMs to normal, and these continued to be of long duration. These results suggest that the initiation of LAMs is modulated by dopamine, whereas the duration of each LAM is Mn sensitive and dopamine insensitive. Importantly the decrease in dopaminergic drive in the absence of dopaminergic neurodegeneration points to specific differences in the pathology of PD versus manganism (Guilarte, 2010) and highlights the relevance of zebrafish larvae as a model system for human manganism.

Taken together, our experiments establish that, in a zebrafish model of manganism, Mn causes functional impairments in sensory, motor and neuromodulatory neurons. These functional impairments are sufficient to explain the postural and locomotor abnormalities that we observed. These individual effects when further probed can direct effective therapies that will ameliorate or cure the symptoms of manganism.

## MATERIALS AND METHODS

### Ethics statement and maintenance of fish

All experiments were performed on zebrafish larvae at 3–7 dpf that had been obtained from wild-type stock. Embryos were incubated at 28°C in E3 medium containing NaCl (5 mM), KCl (170 μM), CaCl_2_ (330 μM) or MgCl_2_ (330 μM), and methylene blue (0.6 μM). Zebrafish were maintained and bred following protocols approved by the institutional animal ethics committee (National Centre for Biological Sciences and Tata Institute of Fundamental Research).

### Reagents and solutions

Unless otherwise noted, all chemicals used were of analytical grade and obtained from commercial sources. Metal chloride stock solutions (100 mM) were prepared in E3 medium. All the stock solutions and treatment solutions were freshly prepared before each study.

### Mn-exposure protocol

Zebrafish larvae (3.5 dpf) were exposed to different concentrations of Mn by adding manganese chloride (MnCl_2_) to the E3 medium in which they were growing. The larvae were transferred to 6-well plates, with a different concentration of MnCl_2_ in each well (*n*=20 for each concentration of MnCl_2_). Control untreated larvae (*n*=20) in E3 medium were used as comparison for every set of experiments. All larvae were incubated at 28°C.

In order to standardize the Mn-exposure levels, the larvae were treated with MnCl_2_ solutions of concentrations varying from 200 μM to 10 mM, and the LC_50_ value was obtained as 2 mM. Control experiments to check the specificity of behavioral changes that occurred upon Mn exposure included similar studies with other biologically essential metal ions. The representative metal ions chosen were Na^+^, Ca^2+^, Mg^2+^, Cu^2+^ and Zn^2+^. In order to negate the effect of anions, all metal salts chosen had the same chloride counter ion. Na^+^, Ca^2+^ and Mg^2+^ solutions were prepared by adding extra metal salts to the E3 medium.

All experiments to study the effect of Mn on zebrafish larvae were performed after 24- to 48-hour exposures to MnCl_2_ solutions (<2 mM) in E3 medium.

### Recovery experiments

Mn-treated larvae (48-hour exposure to MnCl_2_) were washed several times with normal E3 medium and transferred back to E3 medium devoid of Mn. Further experiments on the recovered larvae were performed after 24 to 48 hours of recovery.

### Larval behavior observations

Each Mn-treated larva was transferred to a separate (35 mm) well (of a 6-well plate) containing 10 ml of E3 medium with the identical concentration of Mn to which it had been exposed. Morphological changes and behavioral patterns in both control and Mn-treated larvae were noted. Spontaneous movements in the larva were observed for 3 to 5 minutes, and unusual movement patterns were noted. Responses to vibrational stimuli were observed over a time period of 5 minutes for each larva with tapping on the edge of the well. The Chi-square test was used to estimate the statistical significance in all the observations. Error bars in all plots represent the standard error of the mean (s.e.m.).

### High-speed video recordings

Mn-treated (48-hour exposure to MnCl_2_), control and recovered (48-hour exposure to MnCl_2_ followed by 24 hours in normal E3 medium) larvae were embedded in agarose with a dorsal orientation. A subset of the larvae was exposed to either dopamine (10 μM in E3 medium) or bupropion (200 μM in E3 medium) externally for 30 minutes. These larvae were also embedded in agarose. The agarose surrounding the tail was carefully removed so that the tail was completely free to move, leaving the head and the swim bladder embedded in the agarose. The free trunk of all larvae, except those that had been supplemented with either dopamine or bupropion, were in E3 medium during the high-speed recordings. The dopamine-supplemented larvae were in E3 medium containing 10 μM dopamine during the recordings, whereas the bupropion treated larvae were in E3 medium containing 200 μM bupropion during the recordings. Larval movement was monitored using a Phantom Miro EX4 (Vision Research, Wayne, NJ) high-speed video camera at 300 fps for 20 seconds. Larval responses to vibrational stimuli were monitored by tapping at the edge of the well containing the larvae. Three different points on the larva, head (between the eyes, H), swim bladder (M) and tail (a little above the tip, T), as shown in Fig. 3Ai–iii, were tracked using MATLAB Digitizing tools (Hedrick, 2008). The *x*,*y*-coordinates were obtained for each point, and the body angle was calculated as the angle subtended by lines drawn from H to M and M to T using the following equation in Origin (OriginLab, Northampton, MA):

$$\theta (\deg)=360-\frac{180}{\pi }\cos^{-1}\left[\frac{\left\{\left(H_{x}-M_{x})(T_{x}-M_{x})+(H_{y}-M_{y})(T_{y}-M_{y}\right)\right\}}{\left\{\sqrt{{\left(H_{x}-M_{x}\right)}^{2}+{\left(H_{y}-M_{y}\right)}^{2}}\right\}\times \left\{\sqrt{{\left(T_{x}-M_{x}\right)}^{2}+{\left(T_{y}-M_{y}\right)}^{2}}\right\}}\right].$$

Body angles were calculated in every frame and plotted against time. Because the angle was obtained as a cosine^−1^, the angles reported do not reflect the direction of movement. Hence, all large angle movements (Fig. 3Aiii) were manually assigned as left or right movements by replaying each video recording. For Fig. 3F, the left movements were depicted above a black line representing the resting position (180°) and right movements were depicted below the black line representing the resting position (180°). The time duration of a LAM was computed as the time taken from the initial resting state to move in a single direction and return back to the original position. The frequency and duration of LAMs were computed for all sets of untreated and treated larvae. Statistical significance was estimated using Wilcoxon rank sum test with Bonferroni correction for multiple comparisons and the Kruskal–Wallis one way analysis of variance (ANOVA) for frequency and duration of LAMs, respectively. Wilcoxon rank sum test was used for comparing the duration of short wags in Mn-treated versus control larvae.

### FM-dye labeling

Larvae were rinsed with E3 medium extensively. The washed larvae were then incubated with *N*-(3-triethylammoniumpropyl)-4-(4-(dibutylamino)-styryl) pyridinium dibromide (FM^®^ 1-43 FX dye) for 30 seconds and rinsed several times with E3 medium. The larvae were fixed with 4% paraformaldehyde (PFA) at 4°C overnight. The fixed larvae were washed several times with PBS and upgraded to 87% glycerol. Finally, the larvae were mounted on coverslips with lateral orientation for confocal imaging.

### Whole-mount immunohistochemistry

The antibodies used for the immunostaining experiments were: mouse anti-E-cadherin (E-Cad) antibody (1:100 dilution, BD, catalog number 610182), mouse ZNP-1 (1:100 dilution, ZIRC), mouse anti-tyrosine hydroxylase LNC1 (1:1000 dilution, Millipore, catalog number MAB318), Cy™ 3-conjugated AffiniPure goat anti-mouse IgG (H+L) (1:750 dilution, Jackson ImmunoResearch, catalog number 115-165-146) and Dylight-488-AffiniPure sheep anti-mouse IgG (H+L) (1:500 dilution, Jackson ImmunoResearch, catalog number 515-485-003).

The general procedure was as follows. The larvae were fixed with 4% PFA at 4°C for 12 hours. The larvae were washed several times with 0.1 M PBS containing 0.1% Triton X-100 (PBT buffer). Larvae were incubated for 3 hours at room temperature with 5–10% normal goat serum (NGS) in PBT. The larvae were then incubated with primary antibody in NGS (1% solution in PBT) at 4°C for 12 hours. The incubated larvae were washed with PBT several times and further incubated for 12 hours with fluorophore-coupled secondary antibody in NGS (1% solution in PBT). For labeling neuromasts, Alexa-Fluor-488-conjugated phalloidin (1:40 dilution) was included in the incubation mixture. After washing several times with 0.1 M PBS, samples were upgraded to 87% glycerol in PBS and stored in the same solution until they were imaged. Finally, the larvae were mounted on coverslips with either ProLong^®^ Gold antifade reagent (Invitrogen) or glycerol. The head was mounted in a dorso-ventral orientation for confocal imaging of the larvae immunostained for tyrosine hydroxylase. The eyes and the yolk sac were removed to place the larva in a stable position. For confocal imaging of neuromasts, the larvae were mounted in the lateral orientation.

### Bungarotoxin labeling

ZNP-1-stained larvae were treated with dH_2_O for 5 minutes, followed by ice-cold acetone incubation for 7 minutes and treatment with dH_2_O for 5 minutes. The larvae were then treated with Alexa-Fluor-488-conjugated α-bungarotoxin (1:100 dilution, Molecular Probes, catalog number B-13422) in NGS (5% solution in PBT) for 30 minutes at room temperature. The larvae were washed several times with PBS and upgraded in glycerol. The larvae were decapitated and the trunk was placed in a lateral orientation for imaging motor neuronal connections.

### Confocal imaging

Whole-mount zebrafish preparations mounted on slides were imaged on a ZEISS LSM 710 laser scanning confocal microscope with 63× oil immersion or 40× water immersion objective with 1× zoom, except for the stereocilia which were imaged at 3× and 5× zoom. Excitation and emission wavelengths were set according to the fluorophores used. *z*-series were obtained with a spacing of 1.00 μm for E-cadherin-phalloidin staining, 2.00 μm for FM-dye staining, 1.00 μm for ZNP-1 antibody and α-bungarotoxin staining and 2.26 μm for antibody staining of tyrosine hydroxylase.

### Data analysis for antibody staining studies

Images obtained on the confocal microscope were analyzed using Fiji software (Schindelin et al., 2012). Neuromasts were located in images obtained after staining for E-cadherin by identifying the characteristic rosette arrangement of hair cells. The number of hair cells per neuromast was counted using the cell counter plugin in Fiji. Statistical analysis for the number of neuromasts and the number of hair cells per neuromast was performed using the Wilcoxon rank sum test. The stereocilia of the otic neuromasts were identified as splayed or non-splayed (Fig. 2B) and counted using the cell counter plugin in Fiji. The intensities of staining by ZNP-1 (control and Mn treated) and of tyrosine hydroxylase (control, Mn treated and recovered) in images were analyzed by choosing identical regions of interest (ROI). The background-subtracted intensity values were analyzed for normality using Lilliefors test, and either repeated measures ANOVA (RMANOVA) (for normal distribution) or Freidman tests (for non-normal distribution) were used to estimate the statistical significance. Tyrosine-hydroxylase-immunoreactive cell bodies in different planes in the images of tyrosine hydroxylase staining were also counted using the cell counter plugin. Because the intensities of tyrosine hydroxylase staining in the Mn-treated larvae were significantly lower, the intensities were multiplied by a constant number for cell counting (Fig. 5E). Cell counts for tyrosine-hydroxylase-immunoreactive cells were plotted as bar plots for different regions using Microsoft Excel, and statistical tests were performed using MATLAB (The MathWorks, Natick, MA). Lilliefors test was used to determine the normality of the data distribution. ANOVA was used to estimate the statistical significance for the tyrosine-hydroxylase-immunoreactive cell counts in telencephalon, pretectum and area postrema. Wilcoxon rank sum test with Bonferroni correction for multiple comparisons was performed on the tyrosine-hydroxylase-immunoreactive cell count in the ventral diencephalon region. Error bars in the plots represent s.e.m.

### Extracellular recording of motor patterns

Mn-treated (48-hour exposure to MnCl_2_, *n*=4), control (*n*=4) and recovered (48-hour exposure to MnCl_2_ followed by 48 hours in normal E3 medium, *n*=6) larvae were anaesthetized using 0.01% tricaine in E3 medium. Anaesthetized larvae were pinned through their notochord onto a piece of Sylgard® (Dow Corning, MI, USA) using fine tungsten wire (California Fine Wire, Grover Beach, CA). The larvae were then washed with external saline (134 mM NaCl, 2.9 mM KCl, 1.2 mM MgCl_2_, 10 mM HEPES, 10 mM glucose and 2.1 mM CaCl_2_, 10 μM d-tubocurarine; pH 7.8; osmolarity 290 mmol/kg). The skin of the larva was gently peeled off to expose the muscles. A thin capillary of 1.1 mm internal diameter and 1.5 mm outer diameter was tapered to obtain a thin glass needle using a micropipette pulling instrument (Sutter Instrument Co. Novato, CA). The pipettes were filled with external saline and had resistances in the range 0.7–1.0 MΩ. A silver electrode was inserted into the pipette such that the electrode dipped into the saline. The pipette was positioned close to the myotomal boundary in the mid-trunk region and a mild suction was applied. The suction allowed the muscles and the axons to be drawn up into the pipette and facilitated measurements of action potentials travelling through these axons. Signals were amplified using a Multiclamp 700B (Molecular Devices, Sunnyvale, CA) amplifier in current-clamp mode with an output gain of 10000×. Spontaneous responses in the larvae were recorded for a duration of 2 minutes.

### Analysis of motor patterns

Neuronal signals were observed as rapid spikes followed by intermittent quiescent periods. Spike times were detected off-line using the threshold search option in Clampfit (Molecular Devices, Sunnyvale, CA) and inter-spike intervals (ISIs) were calculated. The ISIs were used to sort spikes into bursts (ISIs <10 mseconds) and episodes (ISIs between 10 and 100 mseconds). The burst period was the time difference between successive burst start times. Burst duration was the time between the start and the end of a burst. The number of spikes per burst, bursts per episode, burst period and burst duration for spontaneous responses were obtained, plotted and statistically analyzed using custom scripts written in MATLAB (The MathWorks, Natick, MA).

Fisher’s test was used to determine the normality of the data distribution. The Kruskal–Wallis test was used to estimate the statistical significance in all the observations. Error bars in the plots indicate s.e.m.

## Supplementary Material

Supplementary Material
