# Supplementary material for: A zebrafish model of manganism reveals reversible and treatable symptoms that are independent of neurotoxicity
Source: Dis Model Mech. 2014 Sep 26;7(11):1239–51. doi: 10.1242/dmm.016683 (PMC4213728; doi:10.1242/dmm.016683)
Supplement: Supplementary Material [file supp_7_11_1239__index.html]

A zebrafish model of manganism reveals reversible and treatable symptoms that are independent of neurotoxicity — Supplementary Material 

# A zebrafish model of manganism reveals reversible and treatable symptoms that are independent of neurotoxicity

## DMM016683 Supplementary Material

**Files in this Data Supplement:**

- **Supplementary Material**
