# Supplementary material for: A zebrafish model of manganism reveals reversible and treatable symptoms that are independent of neurotoxicity
Source: Dis Model Mech. 2014 Sep 26;7(11):1239–51. doi: 10.1242/dmm.016683 (PMC4213728; doi:10.1242/dmm.016683)
Supplement: Supplementary Material [file supp_7.11.1239_DMM016683.pdf]

Supporting Information

A zebrafish model of manganism reveals reversible and treatable symptoms that are independent of neurotoxicity

Subha Bakthavatsalam[†], Shreya Das Sharma[‡], Mahendra Sonawane[§], Vatsala Thirumalai[‡], and Ankona Datta[†]**

† Department of Chemical Sciences, Tata Institute of Fundamental Research, 1 Homi Bhabha Road, Colaba, Mumbai-400005, India

‡ National Centre for Biological Sciences, GKVK, Bellary Road, Bangalore-560065, India

§ Department of Biological Sciences, Tata Institute of Fundamental Research, 1 Homi Bhabha Road, Colaba, Mumbai-400005, India

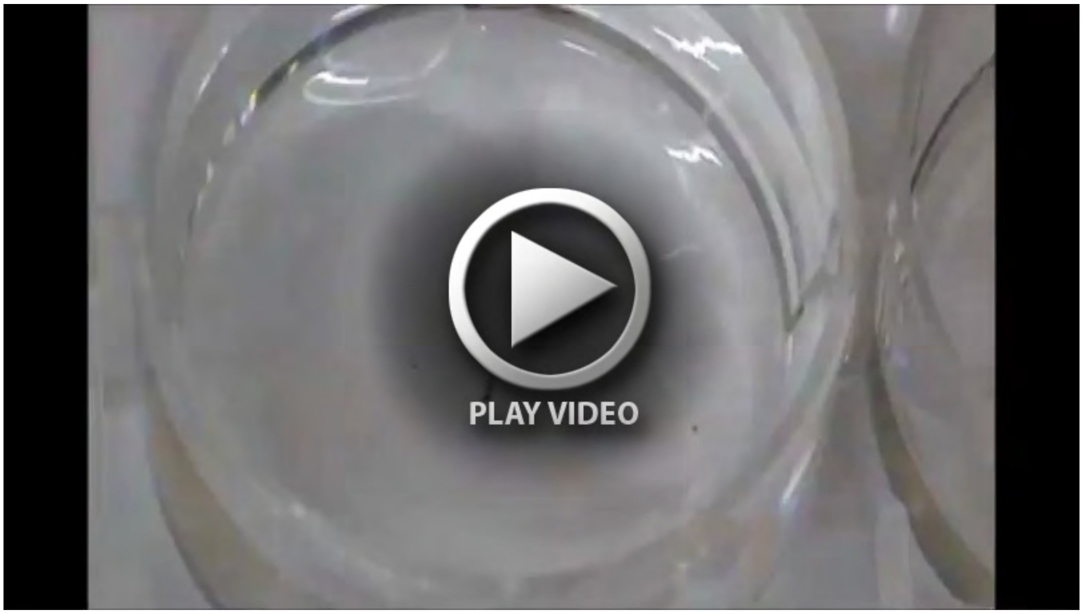

Movie S1: **Circular swimming pattern displayed by 0.8 mM Mn treated larvae (5 dpf) in response to tap at the edge of the well.**

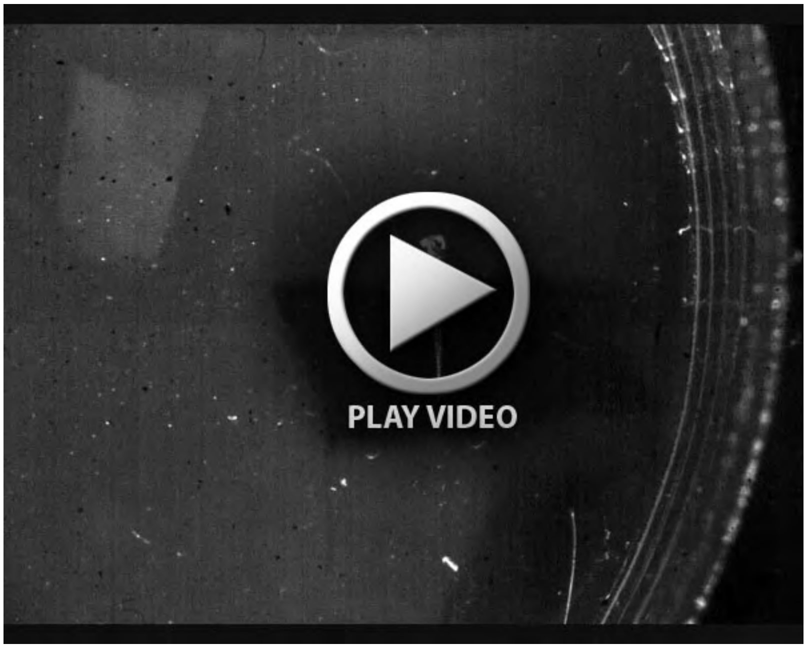

Movie S2: **High speed video recording of agarose embedded larvae.** Wagging movement from a control larvae as seen at 300 fps.

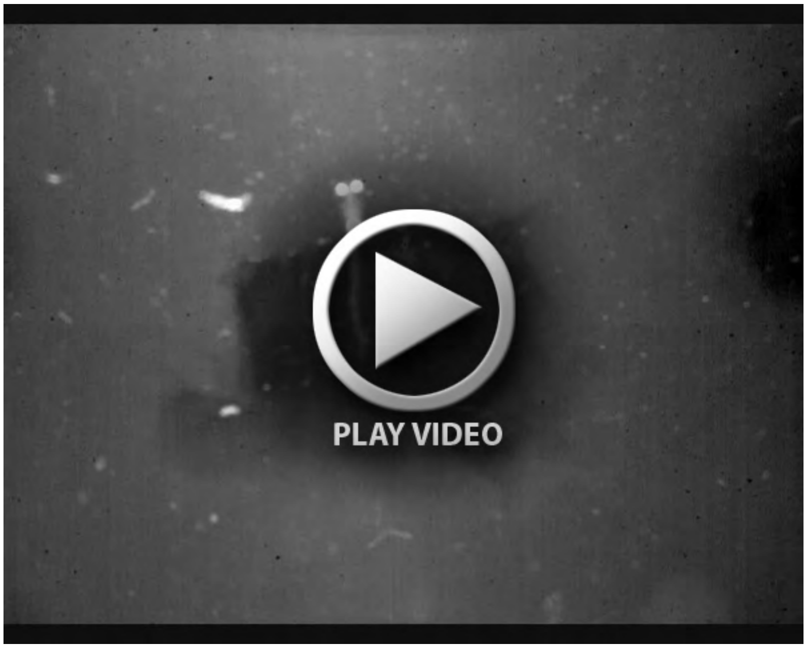

Movie S3: **High speed video recording of agarose embedded larvae.** Large angle movement (two right LAMs in quick succession) exhibited by control larvae as seen at 300 fps.

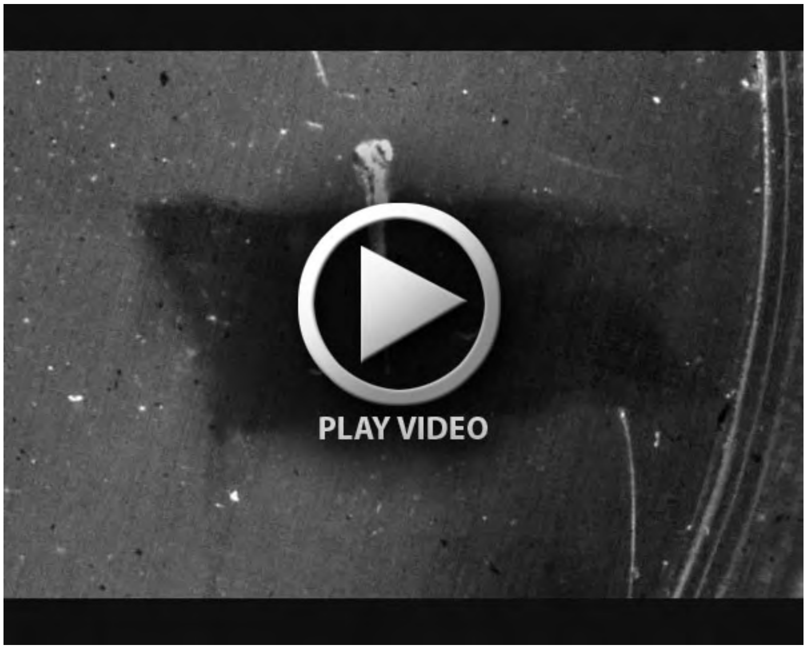

Movie S4: **High speed video recording of agarose embedded larvae.** Large angle movement (one right LAM) exhibited by Mn treated larvae as seen at 300 fps.
